# Dietary Lipid Modulation of Intestinal Serotonin in Ballan Wrasse (*Labrus bergylta*)—*In Vitro* Analyses

**DOI:** 10.3389/fendo.2021.560055

**Published:** 2021-03-23

**Authors:** Angela Etayo, Hoang T. M. D. Le, Pedro Araujo, Kai K. Lie, Øystein Sæle

**Affiliations:** Institute of Marine Research, Bergen, Norway

**Keywords:** gut motility, *in vitro*, lipids, EC-cell, teleost

## Abstract

Serotonin (5-HT) is pivotal in the complex regulation of gut motility and consequent digestion of nutrients *via* multiple receptors. We investigated the serotonergic system in an agastric fish species, the ballan wrasse *(Labrus bergylta)* as it represents a unique model for intestinal function. Here we present evidence of the presence of enterochromaffin cells (EC cells) in the gut of ballan wrasse comprising transcriptomic data on EC markers like *adra2a, trpa1, adgrg4, lmxa1, spack1, serpina10*, as well as the localization of 5-HT and mRNA of the rate limiting enzyme; tryptophan hydroxylase (*tph1*) in the gut epithelium. Second, we examined the effects of dietary marine lipids on the enteric serotonergic system in this stomach-less teleost by administrating a hydrolyzed lipid bolus in ex vivo guts in an organ bath system. Modulation of the mRNA expression from the tryptophan hydroxylase *tph1* (EC cells isoform), *tph2* (neural isoform), and other genes involved in the serotonergic machinery were tracked. Our results showed no evidence to confirm that the dietary lipid meal did boost the production of 5-HT within the EC cells as mRNA *tph1* was weakly regulated postprandially. However, dietary lipid seemed to upregulate the post-prandial expression of *tph2* found in the serotonergic neurons. 5-HT in the intestinal tissue increased 3 hours after “exposure” of lipids, as was observed in the mRNA expression of *tph2*. This suggest that serotonergic neurons and not EC cells are responsible for the substantial increment of 5-HT after a lipid-reach “meal” in ballan wrasse. Cells expressing *tph1* were identified in the gut epithelium, characteristic for EC cells. However, Tph1 positive cells were also present in the lamina propria. Characterization of these cells together with their implications in the serotonergic system will contribute to broad the scarce knowledge of the serotonergic system across teleosts.

## Introduction

The gastrointestinal (GI) tract is considered a complex chemosensory system which can sense changes in gut content and release signals to the enteric nervous system (ENS) (1). The ENS is crucial for modulating gut motility (contractions and relaxations of the muscularis externa) which is required for mixing, thorough digestion of nutrients and elimination of waste products (2). The gut of ballan wrasse (*Labrus bergylta*) is functionally similar to the small intestine of mammals but the evolutionary loss of the stomach led to a different digestive physiology from other vertebrates, including gastric teleosts. The anterior part of the gut of this species has recently been reported as the major site for nutrient digestion and absorption (3, 4) and motility control is pivotal for optimal mixing and digestion of food stuff.

Serotonin (5-hidroxytryptamine, 5-HT) has been recognized for decades as an important signaling molecule in the gut of several vertebrates including fish. Serotonin regulates gastrointestinal motility which affects digestion and feeding behavior in mammals and other vertebrates (2, 5, 6). Several studies show evidences of a similar role of serotonin affecting GI motility in fish (7–11). Disturbances of the gut 5-HT signaling pathway affects motility and can lead to gastrointestinal disorders such as the inflammatory bowel diseases (IBD) in humans (12). There are two sites for production of serotonin within the gut; by a subset of specific epithelial cells called enterochromaffin cells (EC) which accounts for 90% of human 5-HT production, and by enteric serotonergic neurons which produce around 10% of human 5-HT. Biosynthesis of 5-HT requires the conversion of the essential amino acid tryptophan to 5-hydroxytryptophan (5-HTP) by the rate limiting enzyme tryptophan hydrolase (TPH) (13, 14) and the non-rate limiting enzyme dopa decarboxylase (DDC). Mammals and some other higher vertebrates present two TPH isoforms; TPH1 which is found in the intestinal EC cells and TPH2 uniquely found in serotonergic neurons in both the brain and the intestine (13, 15). Both Tph1 and Tph2 have been characterized in fish, some species have two isoforms of Tph1 (16, 17).

EC cells function as sensory transducers by monitoring conditions prevailing in the lumen (nutrients, chemicals or mechanical stimuli) and passing this information to sensory neurons (ENS) innervating the GI tract (10, 18). Recent investigations in humans and mice have identified specific chemosensory receptors in EC cells such as TRPA1 (Transient Receptor Potential Cation Channel Subfamily A Member 1), ADRA2A (Adrenoceptor Alpha 2A) and TRPC4 (Transient Receptor Potential Cation Channel Subfamily C Member 4) that stimulate the production and release of 5-HT from the cytoplasm to the gut mucosa (19–21). Serotonin is then bound to specific receptors of intrinsic primary afferent neurons (IPANs) which activate a cascade of interneurons and motor neurons within the enteric circuitry causing changes in the motility of the GI tract (15). Remaining 5-HT in the pre-synaptic space of the mucosa is then transported into epithelial cells *via* the serotonin transporter (Solute Carrier Family 6 Member 4 - SLC6A4) and metabolized by monoamine oxidase (MAO) (2, 13). Therefore, MAO and SLC6A4 are directly involved in serotonin catabolism. Serotonin is therefore proposed to regulate the contractile activity of the gut by acting as a stimulant and/or relaxing factor of gut motility in vertebrates including some reported teleosts (10, 22). To date, few studies have been conducted on fish addressing the mechanisms by which 5-HT acts along the GI, instead they are focused on the localization of 5-HT positive endocrine and neural cells in the mucosa. Knowledge on the specific role of 5-HT in mediating these effects is still very limited and not fully understood.

At the same time, a similar organization of the development of 5-HT cells within the spinal cord and brain (23, 24) and a similar organization of the enteric nervous system (25, 26) have been reported in teleost and cartilaginous fish. Besides, the organization of the brain serotonergic system in many fish species is considered similar to that in other vertebrates at different levels; for instance, the mRNA expression of enzymes and transporters involved in the serotonergic system is similarly regulated within vertebrates, as well as the strong implication of 5-HT in controlling GI motility (16). It is important to note that although the organization of the 5-HT serotonin system is conserved across the vertebrate subphylum (16, 17), there are differences with mammals regarding the location of 5-HT body cells in the brain and the types of active 5-HT receptors in their guts (10, 17, 27).

The historical use of chemical treatment to cope with sea lice (*Lepeophtheirus salmonis*) in Norwegian Atlantic salmon (*Salmo salar*) farms resulted in the appearance of drug resistant parasites (28) and negative environmental effects (1). Farmed ballan wrasse (*Labrus bergylta*) is a potent candidate to be used as biological control for large-scale delousing of salmon as it is environmentally sustainable and cause no apparent stress to salmon (29–32). Nonetheless, the novel cultivation of this specie faces some challenges such as lack of appetite resulting in high mortality and poor growth (32). Besides the economic interest of this species, its short agastric digestive system offers an ideal model to generate new valuable knowledge as it presents a unique GI anatomy and physiology that greatly differs from mammals and other vertebrates. The aim of this study was to investigate the functionality and regulation of the serotonergic system in the gut of this species by tracing the expression of the main genes implicated in serotonin metabolism following a lipid meal. It was important to first establish the existence of EC cells in the gut epithelium. Second, serotonin in the gut was postprandially quantified to observe its presence and to what extent, and to observe any possible correlation with gene expression. The effect of post-prandial time (starting 10 min following lipid exposure to up to three hours) on both the gene expression and the amount of serotonin present in the gut was analyzed.

An important step to validate the viability of the guts in *invitro* conditions was to observe the modulation of the expression of known genes that are strongly involved in lipid metabolism. For that purpose, fish oil was used as feed in the experiments, expecting it to also stimulate the mechanisms involved in serotonin metabolism. In a general perspective, this study contributes to broaden the knowledge on the digestive system of an a-gastric species, the ballan wrasse, and its enteric serotonergic system.

## Materials and Methods

### Transcriptomic Analysis

The genome (European Nucleotide Archive accession number: PRJEB13687) and intestinal transcriptomes of ballan wrasse (Gene Expression Omnibus accession number: GSE93191), recently available in (3) were analyzed to investigate the serotonergic system. Lie, Tørresen (3) reported the complete set of RNA transcripts (transcriptome) in four different segments of the intestine and their differential expression among the four segments: segment 1 versus (vs) 2, 1 vs 3, 1 vs 4, 3 vs 4 and 2 vs 3. In the present study, by meta-analyzing this intestinal transcriptome [from additional file 6 (3)], we wanted to first demonstrate the presence of 3 groups of genes of interest; 1) genes involved in lipid metabolism, 2) genes involved in serotonin metabolism and 3) genes considered as markers for either EC cells and/or serotonergic neurons.

### Fluorescence Immunohistochemistry

Farmed wrasse (20 - 30 g.) were anesthetized and killed by a blow in the head. Subsequently, the first region of the gut was excised and washed in cold PBS before incubation in freshly made 4% PFA for 24 hours. Tissue was then transferred to a 15% sucrose solution overnight and then 30% sucrose until it sank. Tissue was always kept at 4°C before embedding in OCT and sectioned on cryotome cryostat (-20°C). Sections (10 µm) were mounted on positively charged glass slides (Superfrost^©^; Mentzel, Braunshweig, Germany) and stored at -20°C until further use.

A commercial monoclonal antibody was used to detect 5-HT in the gut (ab66047, Goat Anti 5-HT antibody, 1:800, Abcam). Sections were left at room temperature (RT) overnight, post-fixed in pfa 4% for 5 min and rehydrated twice in trisbuffered saline (TBS) for 5 min. Antigen retrieval was performed by boiling the sections in a microwave submerged in a 10 mM citrate buffer, pH 6,0 at 650 W for 6 min. Sections were washed twice in TBS 0,05% tween for 5 min. Non-specific binding was prevented by treatment with blocking solution (TBS 0,5% bovine serum albumin (BSA) and 0,5% dry milk powder) at RT for 2 hours. The blocking solution was drained, and the sections were incubated with the primary antibody, diluted in TBS 1% BSA at 4°C overnight. The following day, sections were washed twice with TBS 0,05% tween for 5 min and incubated with a secondary antibody, the Alexa594 labelled (ab150132, Donkey Anti-Goat IgG H&L 594, 1:500, Abcam) at RT for 1 hour. This incubation was performed in the dark in a humidified chamber covered with parafilm. The preparations were then rinsed three times with TBS before being mounted with DAPI medium (ab104139, Abcam). They were analyzed the following day and photographed (Nikon Ti-E, Japan).

### Fluorescence *In Situ* Hybridization Using miRNA LNA Probes

Cryosections were prepared as describe above. Double digoxigenin (DIG)- labeled miRCURY LNA™ microRNA detection probes (QIAGEN) were used in this study. *tph1* detection probe (/5DiGN/ACAACTGAAGCTCAACAGTGA/3DiG-N/) together with a positive control (*β-actine* probe:/5DiGN/TACAAGCGATACTACAACCAT/3DiG-N/) and a negative control probe made of random nucleotides (5DiGN/GTGTAACACGTCTATACGCCCA/3DiG-N/) were previously designed. It was important to maintain a RNase-free environment during all steps in the *in situ *hybridization procedure. Sections were left to dry overnight and post-fixed in freshly made pfa 4% for 5 min and rehydrated in PBS for 5 min. Immediately after, sections were treated with proteinase K,15 μg/mL in PK-buffer supplied by the manufacturer (Exiqon, Cat #339450) and incubated for 15 min at RT. Sections were washed twice in sterile PBS for 5 min..

An aliquot of the double-DIG-labeled LNA probe (*tph1*) was firstly heated to 90°C for 4 min using a heating block and diluted to optimum concentration (40 nM) with the formamide-free Exiqo ISH buffer (Exiqon, Cat #90000). 50 uL of the probe mixture were added on the dried tissue sections and covered with a coverslip avoiding air bubbles. Hybridization was started at 54°C for 60 min using an incubator. The same procedure was followed for both negative and positive controls. After incubation, the coverslips were gently removed, and the slides were placed in 5× SSC at RT. A round of stringent washes (5 min each) were performed in pre-heated SSC buffers (54°C) as following: once in 5× SSC, twice in 1× SSC and twice in 0.2× SSC. Slides were then placed in 0.2× SSC at RT for 5 min and transferred to PBS 0.01% tween. From here on, all the steps were carried out in a horizontal humidifying chamber. To avoid unspecific binding, sections were blocked using PBS 0.1% tween, 1% BSA, 1% dry milk for 15 min at RT.

FITC Anti-DIG (ab119349, Mouse Monoclonal to Digoxigenin,1:800, Abcam) was diluted in PBS 0,05% tween,1% BSA and applied to the sections. Incubation was performed at RT for 60 min, away from the light followed by 3 washes in PBS 0,01% tween and 2 washes with distilled water. The preparations were then mounted with DAPI medium (ab104139, Abcam). They were analyzed and photographed the day after (Nikon Ti-E, Japan).

### Fish and Intestine Preparation

Ballan wrasses juveniles were provided by Marine Harvest Labrus (Øygarden, near Bergen, Norway). The facility has general permission to rear all developmental stages of *Labrus bergylta*, license number H ØN0038 provided by the Norwegian Directorate of Fisheries (https://www.fiskeridir.no/English). Fish were nursed in 3 m^3^- tanks in a temperature-controlled room (around 14°C), 24:0 h light: dark photoperiod and fed every 15 minutes with a commercial pellet diet (Skretting, Norway). These are standard rearing conditions for wrasse. Fish weighing 20 - 30 g were transferred from the Marine Harvest farm to the Institute of Marine Research (Bergen, Norway) laboratory and were kept under conditions identical to those at the nursing station for one day prior to the experiments. Overnight fasting was required to ensure empty guts for the trials.

On the day of the trial, the fish were anaesthetized by placing them in a bath with MS222 (30 mg/ml) before being killed with a blow to the head. Eviscerated intestines, including esophagus and anus with the surrounding skin, were immediately immersed in Ringer’s solution according to Rønnestad, Rojas‐Garcia (33, 34). The luminal content was flushed out with a gentle squirt of Ringer’s solution and the intestines were either mounted to a glass tube for the drug treatment in Experiment 1 or administered a nutrient bolus in Experiment 2.

### Experiment 1—Effects of 5-HT on Gut Motility

To investigate the effect of 5-HT on gut motility of ballan wrasse, we qualitatively evaluated the changes in motility patterns of intestines exposed to five different concentrations of 5-HT (16 nM, 160 nM, 1.6 µM, 16 µM, 160 µM). The concentrations were chosen based on previously reported levels of 5-HT in blood plasma and gut tissue of fish (35–37). Firstly, a physiological concentration (16nM) was calculated and adjusted based on the aforementioned reports and the weight of ballan wrasse intestines respectively. The physiological level was set as the minimum concentration treatment with five additional treatments increasing in concentration 10 times each. Serotonin hydrochloride (H9523, Sigma Aldrich) was freshly dissolved in distilled water and immediately added to organ baths containing the intestines immersed in aerated Ringer’s solution. Control replicates were prepared by adding distilled water instead of serotonin hydrochloride. The intestines were carefully stretched out longitudinally inside a regular glass tube (4 cm Ø, 20 cm long) filled with Ringer’s solution, with the oral opening closed and the anus open according to (34). After a 15-min. acclimatization period, each intestine was exposed to one of the five concentrations of 5-HT and a series of time-lapse images of the intestine was recorded for 10 minutes for qualitative analysis of gut motility patterns. The image acquisition and spatial-temporal (ST) map plotting were conducted according to (34). Briefly, the time-lapse series of images was captured on video by a camera (Nikon DS-Fi3) with a macro lens (Nikon, AF Micro-Nikkor 60mm f/2.8D), at a resolution of 1024×768 pixels. The capture was controlled with the NIS-Elements Confocal 4.51.01 software and captured 3.5 frames s^-1^. Video frames for each intestine were extracted at intervals of 1.2 frames s^-1^. Intestinal diameters were measured along the intestines for constructing the ST maps to qualitatively examine change in motility patterns induced by 5-HT compared to the control. For each replicate, six intestines including a control and five intestines exposed to five different doses of 5-HT were processed in parallel in each video. The experiments were run in duplicates.

Intestinal contractions are observed as diagonal repeated patterns in the ST map. The ST map show a narrow part of the intestine as a light shade on the map. The Y-axis of the map show time and the x-axis show the intestine from the anterior end to the posterior. When a narrow part of the intestine (light shade) is moving along the intestine in caudal direction it is manifested as a diagonal line on the heat map. This line shows the length of the contraction (Y-axis) and for how long (Y-axis from top and down). The length and frequency of these patterns show the type of peristalsis in the intestine.

### Experiment 2—Effects of Lipid Sensing on Serotonin and Lipid Metabolism

To elucidate the effects of fatty acids on the expression of genes involved in serotonin and lipid metabolism, we analyzed the expression of a range of genes involved in lipid and 5-HT metabolism (**Table 1**) and the levels of serotonin in the bulbous of the ballan wrasse intestines fed with lipid bolus. The lipid bolus was made by hydrolyzing cod liver oil (Møllers Tran, containing omega-3-fatty acids and vitamin D) according to (34). Briefly, the cod liver oil was incubated with lipase (lipase from *Pseudomonas cepacia* - 62309 Sigma Aldrich, ≥30 U/mg) at 40°C and pH = 8 for 5 h. The lipase enzyme was deactivated by incubating the mix at 80 °C for 2 h. The nutrient bolus was made and stored at – 20 °C for use within a week. The diets were thawed and warmed up at 14 °C before being administered into the intestines.

**Table 1:** Target genes involved in serotonin and lipid metabolism with the primer sequences used for mRNA expression analyses by terms of RT-qPCR.

*Gene*	*Accession #*	*Gene name*	*Primer sequence*	*Amp size*	*Function*
***apoa4***	XM_020650373.1	Apolipoprotein AIV	F: TAGCTTGGAGCCATGAGGGT R:TGCATCAATCAGCCCATCCAT	117	Lipid transport, absorption
***cd36***	XM_020649455.1	CD 36 molecule	F: ACGGAGGGATAAAACGCACA R:TATGCTGTGGTTCCAGGCTC	181	Fatty acid transport
***plin2***	XM_020630537.1	Perliplin2	F: CAGGAGTATGGTCACGAGGC R:TGTAGACGTGTGTGGCAGAG	175	Lipid droplets formation
***slc27a4***	XM_020638063.1	Solute Carrier Family 27 Member4	F: TGCTCGTCGGCTCTTATTCC R:TTGTAGCCGATAAGCTCGCC	120	Uptake of LCFAs
***elovl1***	XM_020650039.1	ELOVL Fatty Acid Elongase 1	F: GAGGAAGCTGAGCAGAGAACT R:ACTGCGTCACCCGTTTATCC	192	Fatty acids elongation
***tph1***	XM_020659153.1	Tryptophan Hydroxylase 1	F:GAGGGACCACGTAGAGGAAGAT R:CCTTCACTAGTCCTCCCACTTC	190	5-HT synthesis
***tph2***	XM_020653578.1	Tryptophan Hydroxylase 2	F: TGAGGCATGCTTTGTCCGAT R:AACGGACGCTTGATCGTCTT	169	5-HT synthesis
***vmat1/slc18a1***	XM_020643271.1	Vesicular Amine Transporter 1	F: ACCATCACCCTCAGACATCC R:AGACGTGTTCAGCGTTTCCA	104	5-HT packaging
***vmat2/slc18a2***	XM_020626697.1	Vesicular Amine Transporter 2	F: CTAGGCGTTGCTTTCTTACCAG R:CCAATGGCAAAACCGACCC	200	5-HT packaging
***mao***	XM_020630593.1	Monoamine Oxidase	F: CAGCTCATCTGCTCCGGAAA R:TTGGCTGCCGGTATTTCCAT	106	5-HT degradation
***htr4***	XM_020637472.1	5-Hydroxytryptamine Receptor 4	F: GGAAATGGGACTCCCTGCATT R:TTAGGGCTATCCGCTTTGGC	115	Post-synaptic 5-HT transport
***htr3a***	XM_020647355.1	5-Hydroxytryptamine Receptor 3A	F: CTGTCCGTTCTGTGGAGGGATG R:ACTGTGCCGTTCCAGAATAACA	176	Post-synaptic 5-HT transport
***slc6a4***	XM_020644581.1	Solute Carrier Family 6 Member 4	F: GTGTCCTGGATTAGGGGCAA R:AAATCACTCATGCCTGGGCT	144	Pre-synaptic 5-HT transport

**Figure 1:**
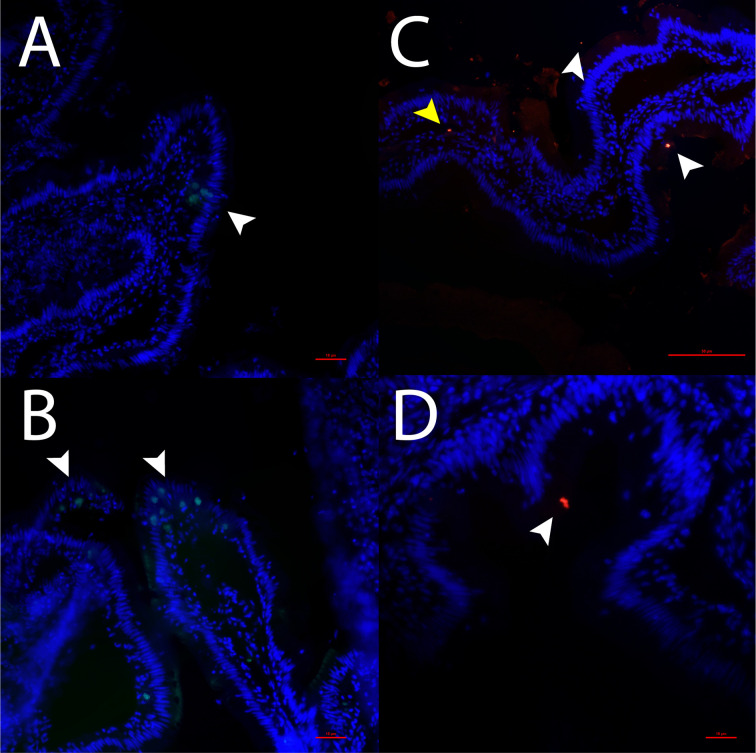
Detection of *tph1* mRNA **(A, B)** and detection of 5-HT **(C, D)** in the gut of ballan wrasse. White arrows in 1A and 1B show *tph1* mRNA positive cells. White arrows in 1C and 1D show 5-HT within the epithelium whereas the yellow arrow in 1C indicates 5-HT expressing cells in the lamina propria. Scale bars are 50 μm in 1A and 1B, and 1C and 10 μm in 1D.

The lipid bolus was administered by injecting a volume of the hydrolyzed fish oil into the bulbous of the extracted intestine. The volume of the nutrient bolus was calculated to mimic ingestion of a meal of 0.1% body weight based on the estimated feed intake of ballan wrasse in the wild (4). The fed intestines were then closed by tying a thread around the esophagus. Intestines were immediately immersed in glass assay tubes filled with 24 ml of Ringer’s solution (pH=7.2 at 14°C) and constant gas flow (95% oxygen + 5% C0_2_) [as described in **Figure 1** in Le, Lie (34)] for an incubation period of 10, 30, 60, 90,120,150 or 180 min following lipid exposure. Incubation time of 3 hours was established to track both short- and long-term responses to the lipid meal. Tissue of the bulbous, the main site for digestion and absorption in ballan wrasse (4), was collected after the incubation periods. The bulbous representing about 40% length of the whole intestine, was cut off and opened by incision; and the gut content was gently washed with Ringer’s solution. Three pieces of tissue (around 50 mg in total) were extracted from three positions along the bulbous and placed in RNA later for further RNA extraction. The remaining tissue from the bulbous was frozen and stored at -80°C for further serotonin extraction. The rest of the gut was discarded. A total number of 42 intestines were analyzed in 6 replicates).

### Gene Analyses by Real-Time RT-qPCR

Total RNA was extracted from the bulbous tissue using a tissue homogenizer and RNeasy Mini Kit (EZ1 RNA Universal Tissue Kit, Qiagen) including a DNAse treatment according to the manufacturer’s protocol. 500-ng aliquots of total RNA were used to generate random hexamer-primed cDNA plate by terms of GeneAmp PCR 9700 (Applied Biosystems, Foster City, UA, USA). Negative controls were performed in parallel by omitting RNA or enzyme. Obtained cDNA was diluted 1:10 before use and stored at -20°C for further use.

PCR primers (**Table 1**) were designed using NCBI design tool (NCBI, Maryland, USA) and Net Primer biosoft. Ribosomal Protein L37 (rpl37) and ubiquitin (ubi) showed a coefficient of variance (CV) < 0.25 and were considered suitable reference genes as previously reported by Sæle, Nordgreen. Gene expression was then determined by means of RT-qPCR using a LightCycler 480 instrument (CFX384™ Real-Time System, Bio-Rad) with the following protocol: pre-incubation at 95°C (5 min.); amplification (40 cycles) at 95°C (10 s); 60°C (10 s); 72°C (15 s); melt curve at 95°C (5 s); 65°C (1 min.); ramp to 97°C (0.11° s^-1^, 5 acquisitions/degree). Each 10 μL DNA amplification reaction contained 2 μL PCR-grade water, 5 μL Lightcycler 480 SYBR Green I Master (Roche Diagnostics), 2 μL 1:10 diluted cDNA template and 0.5 μL (final concentration 500 nM) of each forward and reverse primer. Relative mRNA expression was calculated following normalization to RPL37 and UBI mRNA expression using the ΔΔCq calculation method and the CFX Manager Software (Bio-Rad CFX Manager 3.1).

### Serotonin Extraction and Quantification

Tissue from the 42 intestines (6 replicates with 7 intestines per replicate) that were snap-frozen during the dissections was separately homogenized with buffer (PBS solution 0.02 M, pH=7) to an adjusted final concentration of 80 mg ml-1, homogenized by a TissueLyser (precellys) and further ultra-sonicated on ice using a tissue disrupter (Ultra Turrax, T8/S8N-5G probe, IKA-Werke GMbH, Staufen, Germany). The supernatant was retrieved and assayed by an ELISA analyzer (Serotonin ELISA; Life Span BioSciences, Inc. (LSBio), Seattle) using a target specific pre-coated 96-well plate. A microplate reader was eventually used to measure optical density (OD) of each sample at 450 nm and the obtained OD values were plotted as logarithm against OD values from the standards. The linear equation (y= mx + b) was then used to calculate the concentration of serotonin in the samples. In order to normalize serotonin measurements and minimize the potential method error, the total protein concentration (BCA protein) was assayed for each homogenized tissue by a BCA protein assay according to manufacturer instructions (Pierce, Rockford, IL). The amount of serotonin was expressed in ng µg-1 of total protein content.

### Statistics

The RT-PCR relative normalized gene data for serotonin and lipid metabolism gene targets were normally distributed but exhibited considerable heteroscedasticity, hence weighted regressions (1/σ²) were used to study the gene expression modulation as a function of time as described elsewhere (39). The weighted models were of the form *γ*=*m*×*t*+*b*, where *γ* represents the gene expression, *m* is the slope (aka variation of the gene expression per time unit), *t* is the time in minutes and *b* is the intercept of the model. The adequacy of the regression models was tested by comparing the lack-of-fit to pure error variances at a 95% confidence level. An Excel template developed at Institute of Marine Research that contains the routines for automatic calculation of the weighted regressions and the analysis of their residuals was used. The Excel template is available upon request at HI (par@hi.no). The serotonin concentration measurements were normally distributed and exhibited a high degree of homoscedasticity. One-way Anova test was used to measure significances in the modulation of the serotonin concentrations throughout the feeding trial (p<0.05). R Foundation for Statistical Computing, v2.11.0 (R Development Core Team, Vienna, Austria) was used.

## Results

### Transcriptomic Presence of Genes Involved in Serotonin Metabolism, and Marker Genes for Both EC Cells and Serotonergic Neurons

Intestinal transcripts of ballan wrasse (accession number: GSE93191), available as additional files.6 in (3) were meta-analyzed. First, we confirmed the presence of genes considered relevant for lipid metabolism (*apoa4, cd36, plin 2, slc27a4 and elovl1*) (**Supplementary Table 1**). Genes related to serotonin metabolism and transport (*tph1, ddc, vamt1, tph2, htr4, htr3a, slc6a4*) were also present in intestinal transcripts (**Table 2**). The existence of all these genes involved in lipid and serotonin metabolism in the intestinal transcriptome of ballan wrasse indicated that they were suitable for further expression analysis by RT-qPCR (see results 3.3 and 3.4). Secondly, transcriptome analyses also confirmed the presence of those genes considered as markers for either EC cells and/or serotonergic neurons (*adra2a, trpa1, adgrg4, lmxa1, spack 1, serpina 10*, and *lmx1b*) summarized in (**Table 2**).

**Table 2:** Genes of interest present in the intestinal transcriptome of ballan wrasse.

Gene	Accession number	Segment 1	Segment 2	Segment 3	Segment 4	Pathway
***adra2a***	LABE_00053697	14 ± 8	11 ± 7	15 ± 10	9 ± 4	EC specific, Catecholamine sensitivity
***trpa1***	LABE_00003169	745 ± 155^A^	432 ± 196^A^	262 ± 108^B^	125 ± 39^B^	EC/Neural Cation channel
***adgrg4***	LABE_00020033	1109 ± 456	948 ± 658	1024 ± 197	377 ± 224	EC specific. Transcription factors
***tph1***	LABE_00005595	33 ± 14	21 ± 11	31 ± 8	218 ± 324	EC specific 5-HT synthesis
***ddc***	LABE_00062252	2244 ± 629^AB^	1580 ± 701^A^	1303 ± 496^A^	3666 ± 1315^B^	5-HT synthesis
***vmat1***	LABE_00034664	44 ± 21^A^	42 ± 34^A^	54 ± 14^A^	144 ± 61^B^	EC specific 5-HT vesicular transporter
***lmxa1***	LABE_00030204	23 ± 6	27 ± 10	27 ± 8	19 ± 8	EC specific. Novel regulator of Tph1
***spock1***	LABE_00033390	54 ± 32	54 ± 34	50 ± 19	84 ± 104	EC transcription factor
***serpina10***	LABE_00067292	601 ± 76	536 ± 286	758 ± 179	846 ± 157	EC transcription factor
***lmx1b***	LABE_00014479	121 ± 60	91 ± 56	137 ± 72	44 ± 31	Neural specific regulator of Tph2
***tph2***	LABE_00054456	267 ± 57	238 ± 176	285 ± 58	286 ± 54	Neural specific 5-HT synthesis
***htr4***	LABE_00023438	393 ± 111	373 ± 176	395 ± 95	354 ± 113	Neural 5-HT receptor
***htr3a***	LABE_00003525	1116 ± 326	882 ± 472	1715 ± 373	889 ± 695	Neural 5-HT receptor

Genes expressed by either EC cells, considered as EC-markers, or/and by serotonergic neurons, considered neural markers are shown. Besides, transcriptomic analysis identified differentially expression of the genes among the different segments (from segment 1 to segment 4) along the gut which is shown as the average number of reads per million for each gen in each segment and the standard deviation (SD). Significances in the differential expression among the segments are indicated by letters. 5 intestines were analyzed in the transcriptome, data were retrieved from RNA-sequencing [additional files.6 (3)]. (counts/million) ± SD.

### Fluorescence Immunohistochemistry and *In Situ* Hybridization (Fish) 

The presence of 5-HT and *tph1* mRNA positive cells in the gut of ballan wrasse is shown in **Figure 1**. 5-HT immunoreaction was observed in the apical part of some enteric cells along the epithelium (white arrows in **Figures 1C, D**). Immunostaining of 5-HT was also found in the lamina propria (yellow arrow in **Figure 1C**). To further corroborate the existence of EC cells, the specific localization of *tph1* was assessed using FISH. In contrast to the apical immunolocalization of 5-HT, the expression of *tph1* was observed in the basolateral region of enteric cells (white arrows in **Figures 1A, B**).

### Effects of 5-HT on Gut Motility—A Qualitative Examination

**Figure 2:**
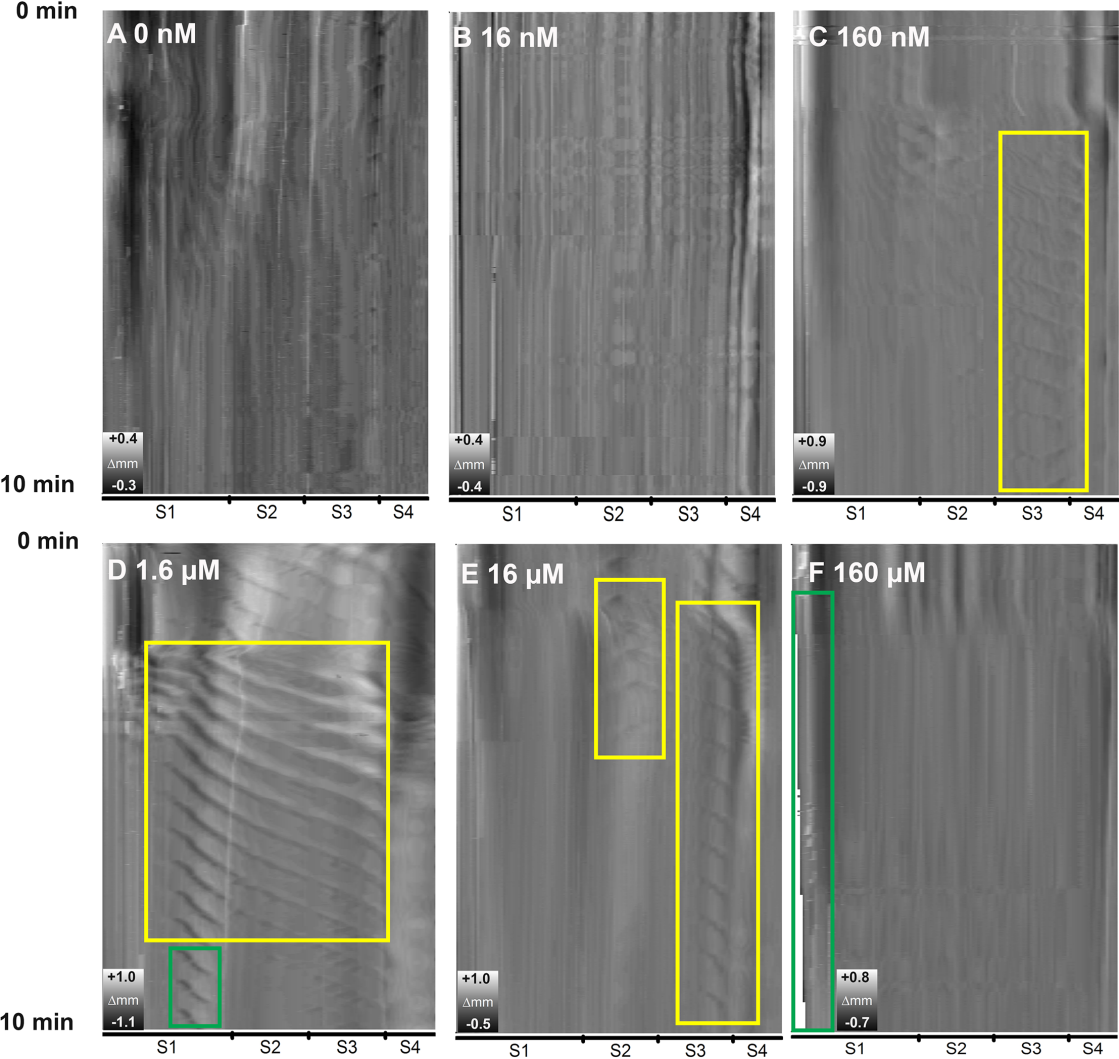
Greyscale heat map showing peristaltic activity as an effect of different concentrations of serotonin on the ballan wrasse intestines. White (light grey) annotates a narrow part of the intestine whereas a darker field, a wider part. The x-axis shows the intestine from the first anterior segment (S1) to the hindgut (S4), the inverted y-axis shows development overt time (0 to 10 min). **(A)** Control without added 5-HT to the medium. **(B–F)** Intestines exposed to 0.016, 0.16, 1.6, 16 and 160 µM 5-HT accordingly. Yellow rectangles show intestinal areas over time where regular peristaltic activity is observed. Green rectangles show how intestines contract length wise, visualized by a narrower grey field at 10 min compared to 1 min. Images are from one replicate. Total number of replicates were 3.

Contractions are observed as diagonal repeated patterns in the grey scale heat map. The heat map registers a narrow part of the intestine as a light shade on the map. When such a narrow part is moving along the intestine in caudal direction (a peristaltic wave) it is manifested as a diagonal line on the heat map. Based on experiments 1, no regular waves of contractions were found neither in control intestines (without 5-HT added to medium) (**Figure 2A**) nor in the lowest exposure (16 nM) 5-HT group (**Figure 2B**). Intestines exposed to 160 nM 5-HT produced ripple waves of contractions in the third segment (the area in the yellow rectangle in **Figure 2C**). Rhythmic and elongated waves together with short waves of contractions (area in yellow and green, respectively in **Figure 2D**) occurred at the posterior part of the first segment, and the entire second and third segment when intestines were exposed to 1.6 µM 5-HT. Intestines exposed to 16 µM and 160 µM 5-HT were less active than those exposed to 1.6 µM 5-HT, as fewer and shorter waves (**Figure 2E**) or no waves (**Figure 2F**) of contractions were observed. The highest concentration of 5-HT (160 µM) induced shortening of the gut length (the green rectangle in **Figure 2F**).

### Differential Expression of Genes Involved in Lipid Metabolism Using RT-PCR

**Figure 3:**
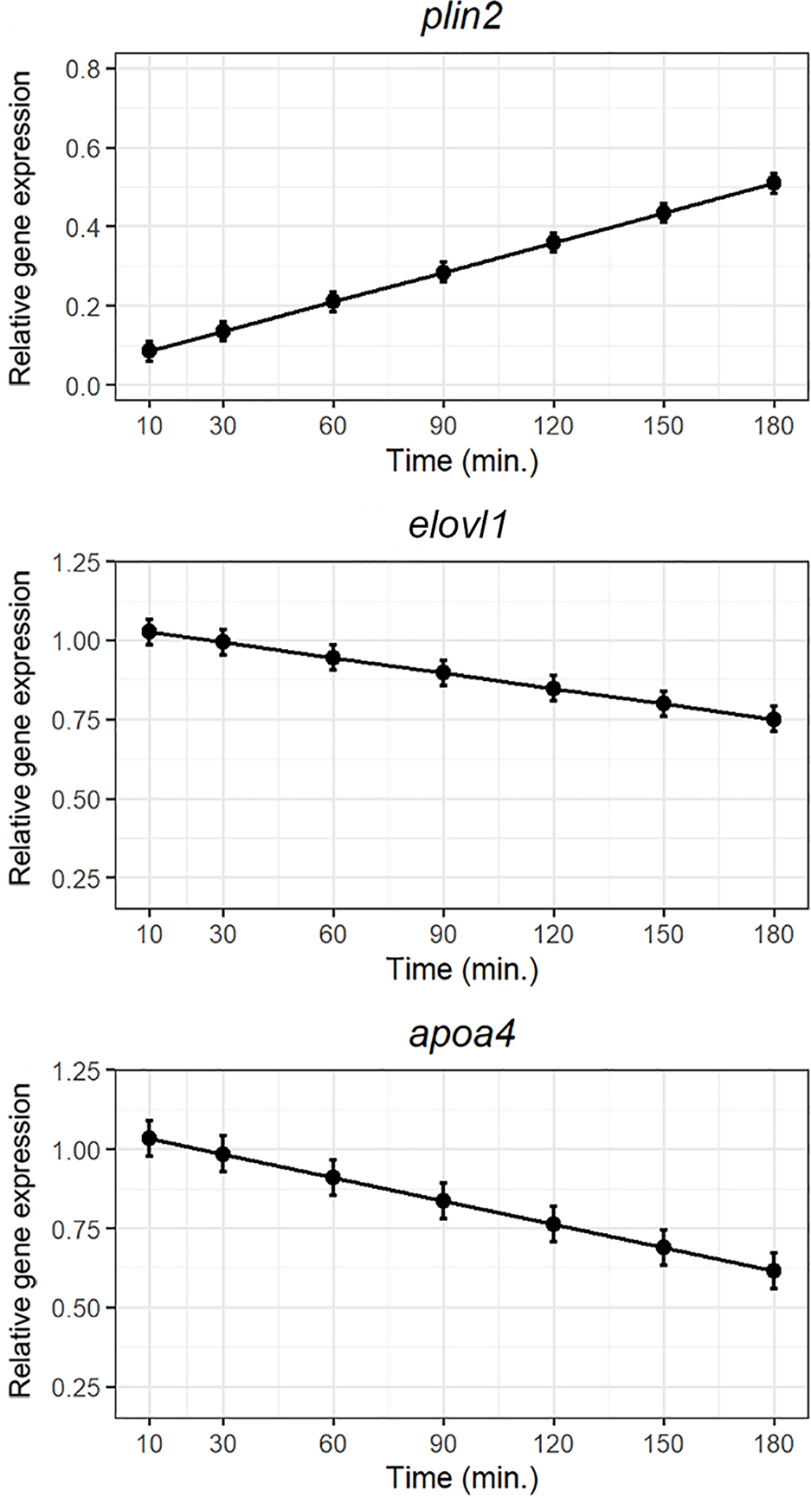
Weighted models for post-prandial relative expression of genes involved in lipid metabolism: *plin2, elolv1*, and *apoa4* with their corresponding standardized residuals. Expression levels were normalized against the reference genes *rpl37* and *ubi*.

The two fatty acid transporters; *cd36* and *slc27a4* were not regulated postprandially (**Supplementary Figure 1**). The important factor of chylomicrons/VLDL; *apoa4*, and the elongase *elovl1* tended to be down regulated and the cytosolic lipid droplet forming protein; *plin2*, showed a tendency to be upregulated following exposure to the lipid bolus as shown in **Figure 3**.

### Differential Expression of Genes Involved in Serotonin Synthesis Using RT-PCR

**Figure 4:**
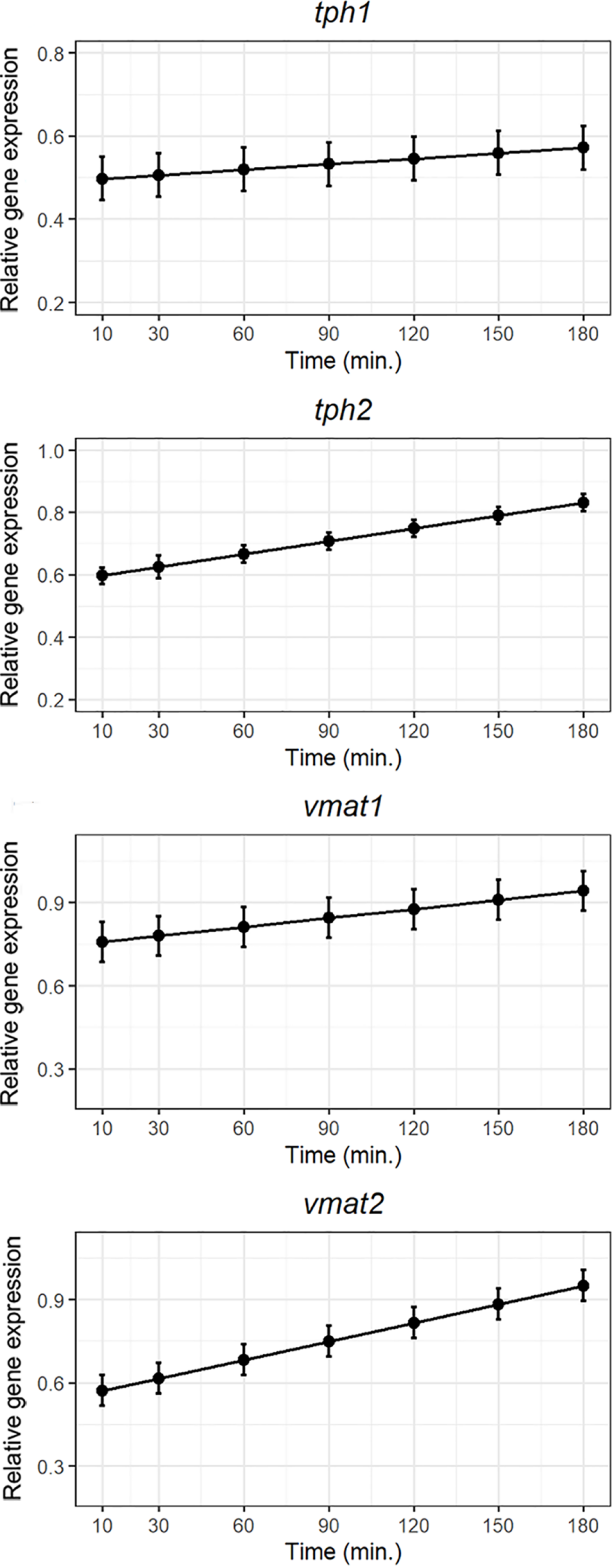
Weighted models for post-prandial relative expression of genes involved in 5-HT synthesis *(tph1* and *tph2*), and the vesicular monoamine transporters (*vmat1 and vmat2*) with their corresponding standardized residuals. Expression levels were normalized against the reference genes rpl37 and ubi.

Both *tph1* and *tph2* mRNA expression increased following exposure to lipid bolus indicating higher synthesis of serotonin within the intestine after a lipid meal. In contrast to *tph1*, the bolus of hydrolyzed lipids tended to strongly upregulate the neural isoform *tph2* mRNA expression (**Figure 4**). Right after 5-HT is produced, there are two vesicular transporters which package newly synthesized 5-HT into granules/vesicles for storage until its release to the gut submucosa. The mRNA expression of both isoforms, the one specific for EC cells (*vmat1*) and the one restricted to neuronal elements in the gut (*vmat2*) increased following exposure which indicates a higher packing of 5-HT 2 to 3 hours after administration of the lipid bolus (**Figure 4**).

### Differential Expression of Genes Involved in Serotonin Catabolism Using RT-PCR

**Figure 5:**
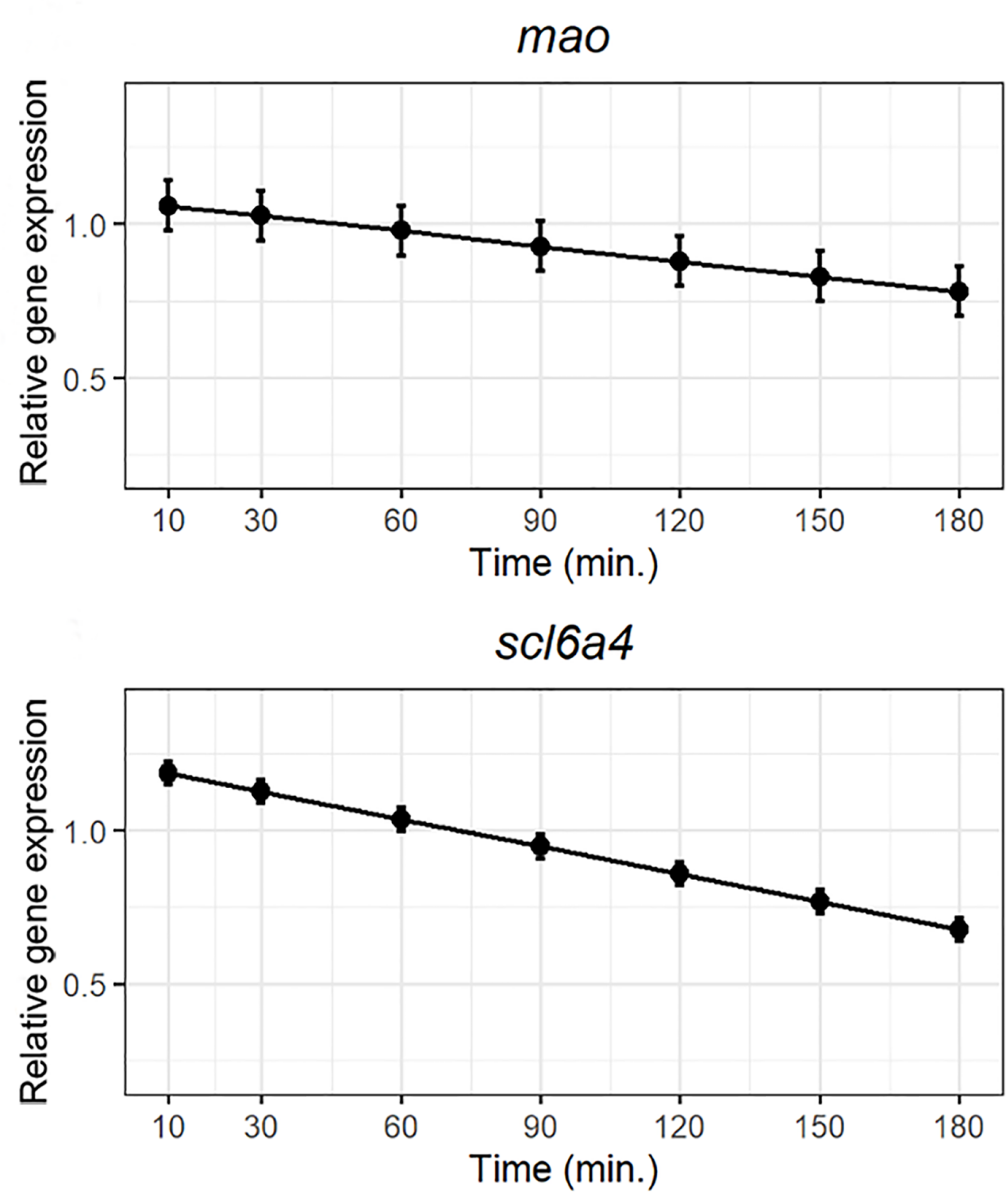
Weighted models for post-prandial relative expression of the monoamine oxidase (*mao*) and the presynaptic 5-HT transporter (*slc6a4*) with their corresponding standardized residuals. Expression levels were normalized against the reference genes rpl37 and ubi.

The 5-HT oxidative enzyme *mao* together with *slc6a4* were notoriously modulated by the lipid bolus (**Figure 5**). Both genes were highly expressed over the first hour post-prandial followed by a gradual downregulation reaching the lowest expression at the end of the trial (**Figure 5**).

### Quantification of Serotonin Levels in Intestinal Tissue and Differential Expression of Its Neural Receptor Genes Using RT-PCR

**Figure 6:**
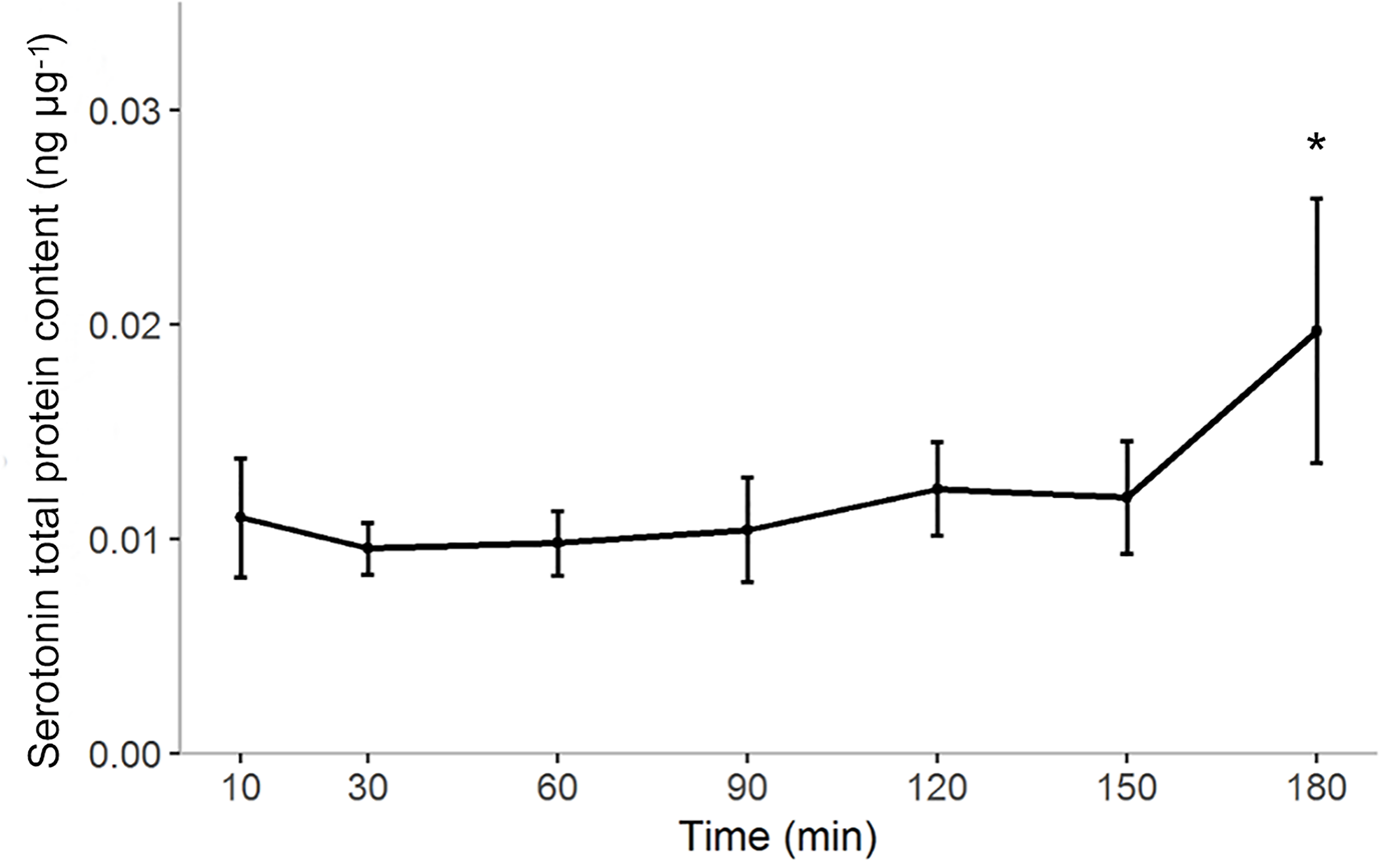
Post-prandial 5-HT concentration in the gut of ballan wrasse expressed as ng of serotonin per µg of total protein content (ng µg-1). Data points indicate means with the corresponding standard deviation (n=6). Significance changes in the concentration of serotonin (p < 0.05) were found between 10 minutes and 180 minutes post-prandial and indicated by *.

Serotonin levels in the bulbous did not show large fluctuations over the first two hours postprandially. However, the amount of serotonin post-prandial increased significantly from 0.011 ± 0.006 (10 minutes post-prandial) to 0.020 ± 0.01 (180 minutes post-prandial) ng µg^-1^ of the total protein content (one-way anova p= 0.004) (**Figure 6**).

No correlation was found between 5-HT in tissue and the expression of any of the two serotonin biosynthetic genes; Pearson’s product-moment correlation test for *tph2* (n=42, r=0.2 and p value=0.22) and *tph1* (n=42, r=-0.32, p value=0.4).

The expression of the two submucosal neural 5-HT receptors htr3a and htr4, were targeted as they are important for initiating gut motility and secretory reflexes. The relative normalized expression of htr3a varied between 0.45 and 0.94 (n=42) and between 0.725 and 0.942 (n=42) for htr4 following exposure to lipid bolus. None showed to be modulated by dietary lipids.

## Discussion

### EC Cells Presence in the Gut of the Stomach-Less Ballan Wrasse

Both serotonergic neurons and EC cells have been characterized within the gut of different teleost species. The presence and location of EC cells in teleosts varies greatly among species, being found both in the stomach and gut, only in the gut (9, 40), or not being present at all as reported in zebrafish (41) and in another stomach-less species, the goldfish (9). This is not the case for another two stomach-less species of flatfish (8) that showed 5-HT positive neurons and EC cells in the intestinal epithelium. The presence or absence of EC cells in the gut of labrid fish has not been previously reported.

Several genes (*ADRA2A, TRPA1, ADGRG4, TPH1, DDC, VMAT1, LMXA1, SPOCK1* and *SERPINA10*) reported as markers for EC cells in higher vertebrates (14, 18, 19, 21, 42) were identified in the genome and intestinal transcriptomes of ballan wrasse. We therefore consider the intestinal transcriptomic expression of these characterizing genes as a good indicator of the presence of EC cells in the gut epithelium of ballan wrasse. *Lmxa1* was reported as essential for the expression of TPH1 in the gut of mice (14). Both *lmxa1* and *tph1* together with the vesicular transporter *vmat1* which is also involved in 5-HT synthesis within EC cells, were expressed in the ballan wrasse intestinal transcriptome. Another EC marker, *TRPA1* was previously reported to be highly expressed in human and rats EC cells (21) where it actively regulates 5-HT release and further gut contractions. The transcriptome of ballan wrasse indicated high levels of *trpa1* transcripts in the intestine. However, *Trpa1* is not exclusively expressed in EC cells as it has been found in sensory neurons in mice, but at lower levels (43). Therefore, it is a marker for 5-HT producing cells but cannot distinguish between EC cells or 5-HT producing neurons.

Human leukocytes express serotonergic genes such as *TPH1, VMAT1,MAO*, and serotonin receptors (44). Whether fish leukocytes express genes associated with 5-HT synthesis has not been investigated yet and therefore, it is uncertain whether the presented transcriptomic analyses of some of the EC cells markers (*tph1, vmat1, ddc, mao* and *lmxa1*) in the gut of ballan wrasse are exclusively identifying EC cells or if they could also indicate a potential leukocyte 5-HT machinery.

However, the demonstration of 5-HT positive cells in the lamina propria as well as in the apical part of the intestinal epithelium presents evidence of both serotonergic neurons and EC cells in the wrasse gut. Even though Tph1 has been shown to be specific for enterochromaffin cells (13, 22, 45), cultured human T cells (46) and calves circulating leukocytes (47) can also express genes involved in the serotonin machinery i.e., *TPH1*. Both authors conclude that mammalian leukocytes can produce and store serotonin in response to stimuli. To our knowledge, neither *tph1* nor other genes involved in the serotonergic system have been reported in leukocytes from any fish species, but it is likely based on the aforementioned evidence. Wrasse Tph1 positive cells were identified in the intestinal epithelium as well as in the lamina propria. The epithelial *tph1* expressions are found in the basolateral part of the cells. mRNA distribution in fish enterocytes or associated cells happen in unchartered areas, but in mammals it has been shown to be expressed in both the apical and basolateral part of the cell (48). The sum of transcriptomic analysis together with the exact localization of 5-HT and Tph1 expressing cells present compelling evidence for the presence of EC cells in the ballan wrasse.

### Serotonin Is Involved in Gut Motility

The present study confirmed that serotonin affected gut motility in ballan wrasse intestine. This finding is in agreement with previous studies in fishes for which serotonin has also been found to evoke intestinal contractions and regulate intestinal motility in goldfish (*Carassius auratus*) (10) and tilapia (*Oreochromis mossambicus*) (9) *in vitro.* The involvement of serotonin in gut motility has been confirmed in mammals (5, 49–52). It is hypothesized that 5-HT modulates gut motility through the gut neural receptors *Htr3a* and *Htr4* (5, 53, 54) which were expressed in the intestine of ballan wrasse.

### Post-Prandial Lipid Metabolism in the Gut of Ballan Wrasse

The *in vitro* “meal” used in these experiments was cod liver oil, rich in omega-3 polyunsaturated fatty acids. Fat is a strong stimulus for the modulation of the gastrointestinal function resulting in an increase of contractile activity as reported in humans (55). In wrasse, stretch exercised on the intestine by a bolus (“meal”) is a strong driver for peristaltic movements (34). To ensure the viability of the system we investigated the post-prandial expression of lipid metabolism associated genes. High-lipid meals triggers upregulation of *apoa-IV* in fish (56, 57), as was demonstrated in the current study as well as in mammals (58). The up-regulation of *apoa-IV* was immediate posterior to the administration of the hydrolyzed lipids, peeking 30 to 60 min postprandially. Intriguingly, *plin2* coding for a protein that is essential for the formation of cytosolic lipid droplets, showed a steady increase after the *apoa-IV* had peaked. We have previously demonstrated that perliplins are upregulated in response to obstruction of chylomicron formation with consequently arrested lipid transport out of the enterocyte (57). We hypothesize that the *in vitro* intestine lacking a circulatory system, does not transport lipid particles out of the enterocytes, and consequently store absorbed lipids in cytosolic lipid droplet.

The fatty acid elongation (*Elovl1)* activity has been reported to be inhibited in livers of rats (59) and contradictory stimulated (60) by fish oil-enriched diets. The expression of *elovl1* mRNA in ballan wrasse decreased significantly postprandially.

It is important to remark that the studied lipid genes are known to be strongly involved in lipid metabolism. The observed modulation of lipid metabolism associated genes demonstrate the presence of active cellular mechanisms that efficiently respond to nutrients. This was important to be confident about our method and reliably describe the serotonergic system. According to Bellono, Bayrer (19), EC cells have receptors and transduction mechanisms that detect ingested chemicals and contribute to other sensory or neural signaling systems. Our results validated the viability of the intestines *in vitro*, and therefore, the implied viability of EC cells in the intestine of ballan wrasse.

### The Source of Enteric Serotonin: The Role of *tph1* and *tph2*

The mechanisms by which serotonin production is stimulated in serotonergic enteric neurons together with the role of neural 5-HT in the gut remains unclear. For decades, it was widely believed that serotonin was mainly produced by TPH1 in EC cells and the contribution of the enzyme TPH2 (neural form) in the production of serotonin in the gut was substantially smaller and not very clear (22, 61). Li, Chalazonitis (6) knocked out *Tph1* and *Tph2* in mice and they observed that the deletion of *Tph2* had severe implications in gut motility whereas *Tph1* deletion did not interfere with the constitutive gut motility. The latter was also observed by Yadav, Balaji (62). Here we demonstrate the presence of EC-cells in ballan wrasse intestine. We also know that nutrients such as lipids modulates peristalsis (34). The expression of *tph1* in ballan wrasse was not strongly regulated by lipid-rich meal whereas its neural isoform, *tph2*, was considerably upregulated postprandially. Wrasse *tph1* was expressed throughout the whole trial showing a tendency to be upregulated with time. We then suggest that 1) *tph1 is* involved in the production of 5-HT in the gut throughout the whole trial (from 10 up to 180 min postprandially) and (2) at least part of the observed mRNA *tph1* is contained in EC cells. Although EC cells can be activated by stimuli and respond by releasing 5-HT (18), our results did not clearly demonstrate a correlation between mRNA *tph1* and post-prandial time. Tph1 positive cells were also observed in the lamina propria by terms of *in situ* hybridization, accounting for an unknown part of the mRNA quantification. Although it is tempting to assume that Tph1 positive cells in the lamina propia of ballan wrasse might correspond to leukocytes as in mammals (46, 47), the origin in fish is unknown and requires further research. On the other hand, *tph2* demonstrated a stronger correlation post-prandial time than *tph1*. In this regard, we suggest a more significant role of Tph2 (in serotonergic neurons) at producing serotonin in response to feed compared to Tph1.

As previously mentioned, *Lmx1a* is a novel EC marker essential for the production of TPH1 in intestinal EC cells in mice (14) whereas its paralog, *Lmx1b* regulates serotonergic neuron development in the brain of mice (63) and is essential for the regulation of TPH2 in serotonergic neurons (64). Both genes, *Lmx1a* and *Lmx1b* are part of the Lim homeobox (Lhx) gene family which encodes transcription factors that have been conserved in evolution. Makarev and Gorivodsky (65) reported *Lmx1b* to have the lowest expression level among many others Lhx genes in the intestine of mice. In addition, they also reported that the level of some of the Lhx genes transcripts were more than nine to fourteen times higher than that of *Lmx1b*. This extremely low expression of *Lmx1b* relative to *Lmx1a* in the intestine of mice was concluded to explain higher TPH1 enzyme activity compared to TPH2 activity (14). However, in the ballan wrasse intestine the roles are reversed. *Lmx1b* transcript levels were more than 4 times higher than *lmx1a* pointing to higher Tph2 activity compared to Tph1.

There are contradictory reports on the role of both EC cells and enteric neurons (61). Our findings in this stomach-less fish species point to a superior role of serotonergic neurons (*tph2*) than EC cells (*tph1*) in the regulation of 5-HT stimulated by dietary lipid.

### Catabolism of Serotonin

Serotonin released in the submucosa is eventually transported into epithelial cells *via* SLC6A4 (SERTs) transporters and catabolized by two monoamine oxidases; MAO A and MAO B in mammals (18). However, in teleosts, these oxidases do not seem to fit into the classical Mao A/Mao B binary classification (66) as studies show that mao is present in a single form in several species examined (67). In concordance, *mao* was present as a single form in ballan wrasse.

The serotonin transporter SLC6A4, also referred as 5-HTT or SERT, is a major modulator of serotonergic neurotransmission as it is responsible for the uptake of 5-HT from the submucosa to neighboring EC cells for metabolization, determining the magnitude and duration of postsynaptic responses to 5-HT (68, 69). Polymorphism of the human SLC6A4 has been reported as the main cause of gut motility and appetite regulation disorders (45, 70, 71). Our results showed a post-prandial decrease of *slc6a4* suggesting a decreased uptake of total 5-HT. The fact that slc6a4 and *mao* follow the same downward expressional trend postprandially suggests that the rate of 5-HT catabolism peaks the first hour postprandially, probably triggered by food intake (lipid bolus in this study). These results indicate that high abundance of pre-prandial submucosal 5-HT can be quickly degraded after stimulation by dietary lipid. However, pre-prandial levels of 5-HT in the submucosa have not been determined in the present study and the origin and storage of 5-HT in unstimulated guts at the beginning of the trial remains unknown. Between one to three hours post-prandial, there was a down-regulation of the genes responsible of 5-HT catabolism (slc6a4 and *mao*) indicating lower catabolism of 5-HT. This together with the observed post-prandial up-regulation of tph2, would indicate the accumulation of 5-HT in the submucosa long time after food has entered the gut (3 hours post-prandial) which can potentially follow two different paths: either binding to platelets and leave the gut *via* the circulatory system or binding to neural receptors affecting gut motility (18).

Neural serotonin receptors are widely expressed by intrinsic primary afferent neurones (IPANs) which are present in both the submucosal and myenteric plexuses in the gut. There are 5 known receptor families (5-HT1, 5-HT2, 5-HT3, 5-HT4, and 5-HT7) directly involved in gut functions (22). However, 5-HT1p, 5-HT4 and 5-HT3 subtypes have been most extensively studied as they are more implicated in initiating peristalsis and secretory reflexes by luminal 5-HT (5, 72, 73). HTR4 and SLC6A4 have been reported to show a strong correlation in mice intestines having a synergetic activity at regulating the relative abundance and biological activity of 5-HT (74). However, no such a correlation was found in ballan wrasse. The lack of post-prandial modulation of *htr4* and *htr3a* in the gut of ballan wrasse does not mean the lack of activity of these neural receptors. Besides, there might be other receptors with a more relevant role at regulating 5-HT homeostasis in the gut of ballan wrasse as reported for goldfish which 5-ht4 and 5-ht7 receptors were crucial at regulating the contractile activity of the foregut (10).

### Post-Prandial Expression of Serotonin in the Gut of Ballan Wrasse: Modelling the Effect of Lipid Meal in the Serotoninergic System of an Agastric Fish Species

**Figure 7:**
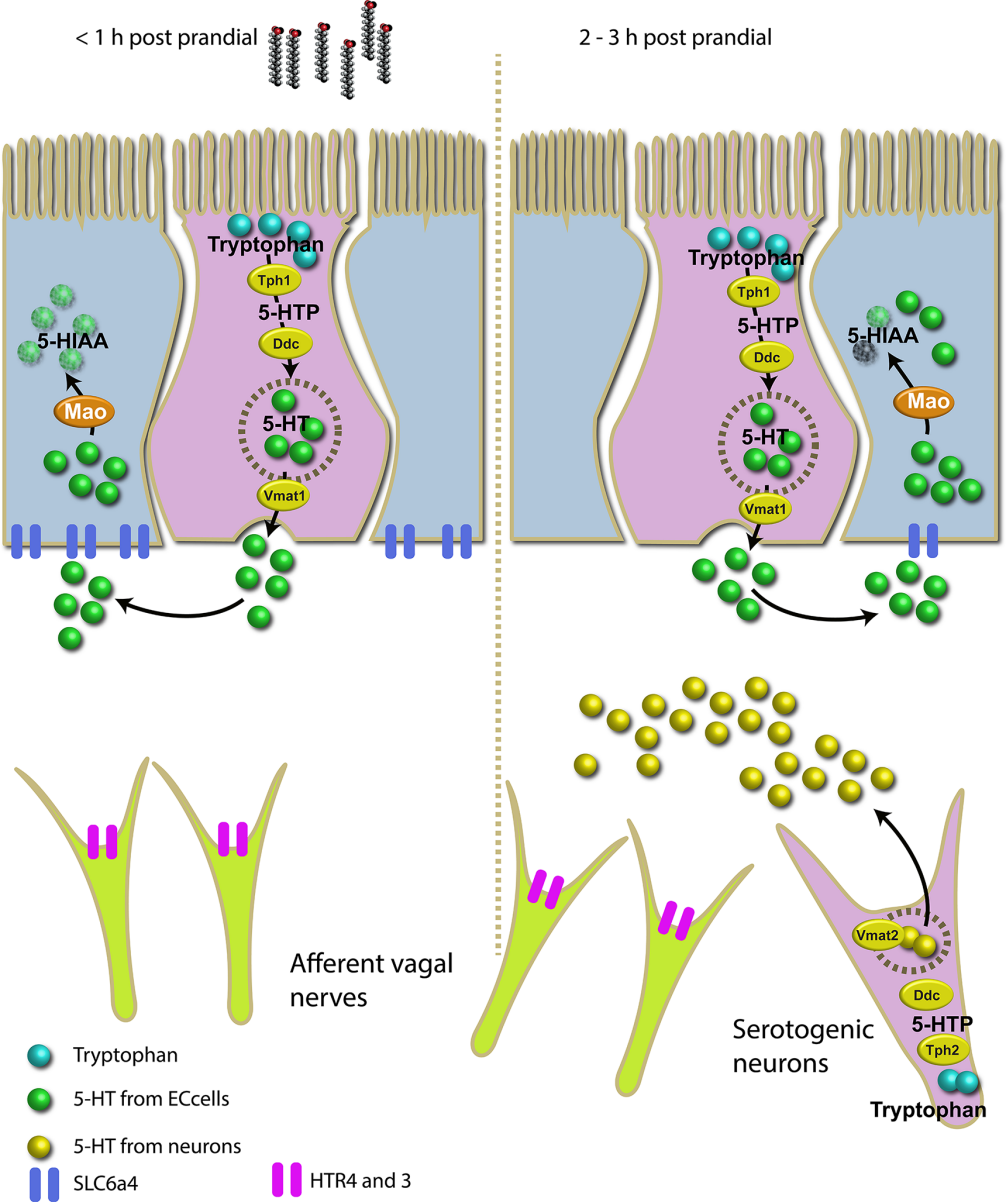
Schematic overview of the proposed molecular mechanism for 5-HT metabolism in intestinal EC cells and serotonergic neurons in a stomach-less specie, ballan wrasse, triggered by a lipid-rich meal. In the left, the serotonergic system 1 hour post-prandial where Mao and Slc6a4 actively metabolize 5-HT which is likely produced by EC cells. In the right, the serotonergic system 2 to 3 hours post-prandial where serotonergic neurons actively produce 5-HT in response to lipid meal. Mao and Slc6a4 remain less active than 1 hour post-prandial indicating the accumulation of 5-HT in the submucosa.

Results showed a considerable increase in the total amount of serotonin post-prandial in the gut of this wrasse species. The higher level of 5-HT post-prandial suggests the presence of a regulatory mechanism that triggers the synthesis of 5-HT and its homeostasis in response to a lipid-rich meal. The proposed genetic mechanism for post-prandial 5-HT metabolism in intestinal EC cells and serotonergic neurons in ballan wrasse is represented schematically in **Figure 7**.

Ballan wrasse lack a stomach and has a different feeding pattern than a lot of species with a stomach. As a typical gastric species would eat a large meal, filling up the stomach before chyme enters the intestine, a wrasse will eat smaller amounts more frequently (4). It is possible that the “grazing” like feeding habits of wrasse therefore does not lead to a burst of response in the intestine. How this system is in species with a stomach remains to be investigated. The diversity of gastric-intestinal physiology in fishes is immense, exemplified by the altered role of CCK in wrasse compared to gastric fish. In wrasse, CCK increase peristalsis towards the anterior intestine for better digestion as opposed to propelling chyme towards the hind-gut in gastric fish (34).

Wrasses are notorious picky eaters and gets quite stressed when handled, for instance when tube fed. To minimize variation due to the fishes experience to stress, ex vivo organ culture was the preferred method. *In vitro* guts of ballan wrasse have been observed to maintain their peristaltic movements up to 14 hours under *in vitro* incubation (34). Serotonin has been proposed to be an important molecule at regulating gut motility [see review by Gershon and Tack (13)] and facilitating segmentation which is an important process in nutrient sensing and absorption (75). Here we have focused on the physiology of the serotonergic system rather than its implications and whether the increase in post-prandial levels of 5-HT is directly linked to the prolonged gut motility of ballan wrasse is still unknown.

## Conclusions

We demonstrate the presence of EC cells in the gut of ballan wrasse. Our results suggest that the enteric serotonergic system is genetically regulated by dietary lipids as suggested in **Figure 7**, where serotonergic neurons (with its neural isoform Tph2) might be the major mechanism involved in long-term 5-HT production after a lipid-rich meal. Tph2 and not Tph1 is proposed to be directly responsible for the substantial increment of 5-HT which might aid the long and maintained motility of the gut eventually affecting digestion of nutrients and appetite regulation of this stomach-less species.

There are obvious anatomical and functional differences in the organization of the serotonergic system of fish compared to that in mammals and other vertebrates. Further research should aim to understand specific functions and mechanisms of the 5-HT system in teleosts.

## Data Availability Statement

The datasets presented in this study can be found in online repositories. The names of the repository/repositories and accession number(s) can be found below in: https://www.ncbi.nlm.nih.gov/geo/query/acc.cgi?acc=GSE93191 and DOI: 10.1186/s12864-018-4570-8.

## Ethics Statement

Ethical review and approval were not required for the animal study because Ballan wrasse juveniles were supplied by a commercial fishfarm (Marine Harvest Labrus, Øygarden, outside Bergen, Norway). The fish was reared in accordance with the Norwegian Animal Welfare Act of 12 December1974, no. 73, §§22 and 30, amended 19 June 2009.The facility has a general permission to rear all developmental stages of Labrus berggylta, license number H ØN0038 provided by the Norwegian Directorate of fisheries (https://www.fiskeridir.no/English).

## Author Contributions 

AE, KL, and ØS: planning and preparing experiment. AE, HL, KL, and ØS: participated in carrying out experiment. AE, HL, KL, PA, and ØS: analysis. AE, HL, KL, PA, and ØS: writing draft version. AE, HL, KL, PA, and ØS: editing. All authors contributed to the article and approved the submitted version.

## Funding

The work was funded by the Research Council of Norway (Project; Intestinal function and health ballan wrasse. Grant no. 244170) and the Institute of Marine Research (Project; Development of the immune system in ballan wrasse – nutritional impact. Grant no. 15465). The funders had no role in study design, data collection and analysis, decision to publish, or preparation of the manuscript.

## Conflict of Interest

The authors declare that the research was conducted in the absence of any commercial or financial relationships that could be construed as a potential conflict of interest.
