# Supplementary material for: Dietary Lipid Modulation of Intestinal Serotonin in Ballan Wrasse (Labrus bergylta)—In Vitro Analyses
Source: Front Endocrinol (Lausanne). 2021 Mar 23;12:560055. doi: 10.3389/fendo.2021.560055 (PMC8021958; doi:10.3389/fendo.2021.560055)
Supplement: Supplementary file 1 [file DataSheet_1.docx]

#### **Supp. figure.1.** Weighted models for post-prandial relative expression of genes involved in lipid transport: *slc27a4* and *cd36* with their corresponding standardized residuals. Expression levels were normalized against the reference genes rpl37 and ubi.

#### *
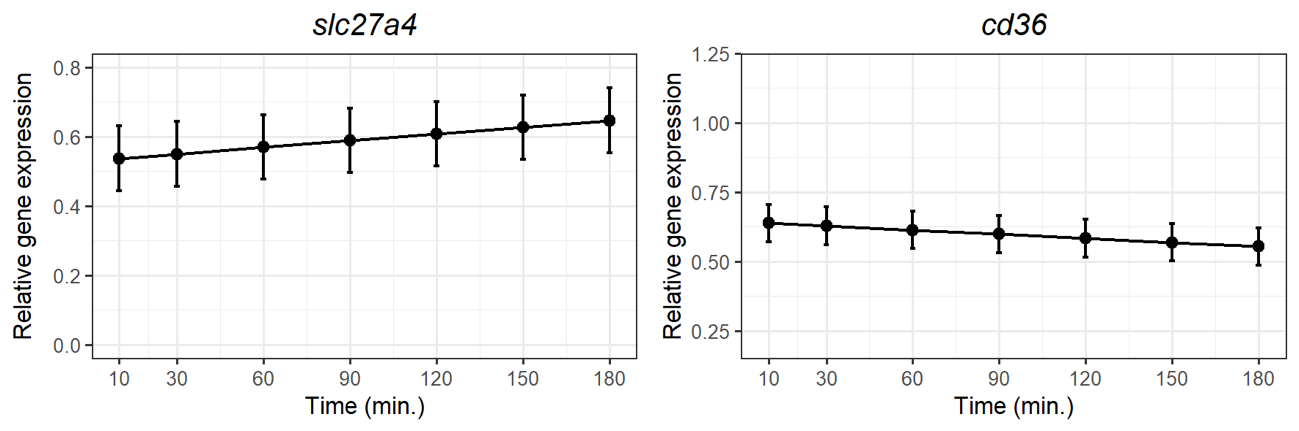
*
