# Supplementary material for: Dietary Lipid Modulation of Intestinal Serotonin in Ballan Wrasse (Labrus bergylta)—In Vitro Analyses
Source: Front Endocrinol (Lausanne). 2021 Mar 23;12:560055. doi: 10.3389/fendo.2021.560055 (PMC8021958; doi:10.3389/fendo.2021.560055)
Supplement: Supplementary file 2 [file Table_1.docx]

**Supp. table.1.** Genes of interest present in the intestinal transcriptome of ballan wrasse. Genes considered relevant for lipid metabolism are shown. Transcriptomic analysis identified differentially expression of the genes among the different segments (from segment 1 to segment 4) along the gut which is shown as the average number of reads per million for each gen in each segment and the standard deviation (SD). 5 intestines were analyzed in the transcriptome, data were retrieved from RNA-sequencing (additional files.6 ([Lie et al., 2018](#_ENREF_31))). (counts/million) ±SD.

| Gene | Accession number | Segment 1 | Segment 2 | Segment 3 | Segment 4 | Pathway |
| --- | --- | --- | --- | --- | --- | --- |
| *apoa4* | LABE_00076034 | 27337±11564 | 29022±15741 | 10868±7594 | 2745±1830 | Chylomicron assembly |
| *cd36* | LABE_00046348 | 50587±20173 | 58589±27430 | 59150±12557 | 3913±6116 | Regulation of fatty acid transport |
| *plin 2* | LABE_00009890 | 14006±5710 | 18248±8286 | 5818±2847 | 1099±520 | Lipid storage droplets |
| *slc27a4* | LABE_00024491 | 2275±693 | 3134±1132 | 2358±806 | 829±159 | Fatty acid transport |
| *elovl1* | LABE_00047458 | 4820±679 | 3772±1247 | 3271±621 | 4765±1324 | Fatty acid elongation |
